# Nationwide randomised trial evaluating elective neck dissection for early stage oral cancer (SEND study) with meta-analysis and concurrent real-world cohort

**DOI:** 10.1038/s41416-019-0587-2

**Published:** 2019-10-15

**Authors:** Iain L. Hutchison, Fran Ridout, Sharon M. Y. Cheung, Neil Shah, Peter Hardee, Christian Surwald, Janavikulam Thiruchelvam, Leo Cheng, Tim K. Mellor, Peter A. Brennan, Andrew J. Baldwin, Richard J. Shaw, Wayne Halfpenny, Martin Danford, Simon Whitley, Graham Smith, Malcolm W. Bailey, Bob Woodwards, Manu Patel, Joseph McManners, Chi-Hwa Chan, Andrew Burns, Prav Praveen, Andrew C. Camilleri, Chris Avery, Graham Putnam, Keith Jones, Keith Webster, William P. Smith, Colin Edge, Iain McVicar, Nick Grew, Stuart Hislop, Nicholas Kalavrezos, Ian C. Martin, Allan Hackshaw

**Affiliations:** 1 Barts Health NHS Trust, London, UK; 2 Saving Faces—The Facial Surgery Research Foundation, London, UK; 3 Barking, Havering and Redbridge University Hospitals NHS Trust, Romford, UK; 4 Brighton and Sussex University Hospitals NHS Trust, Brighton, UK; 5 Royal Free London NHS Foundation Trust, London, UK; 6 Portsmouth Hospitals NHS Trust, Portsmouth, UK; 7 The Pennine Acute Hospitals NHS Trust, England, UK; 8 Aintree University Hospital NHS Foundation Trust, Liverpool, UK; 9 Royal Surrey County Hospital NHS Foundation Trust, Guildford, UK; 10 St George’s University Hospitals NHS Foundation Trust, London, UK; 11 University Hospital of South Manchester NHS Foundation Trust, Manchester, UK; 12 NHS Forth Valley, Stirling, UK; 13 Luton and Dunstable Hospital NHS Foundation Trust, Luton, UK; 14 City Hospitals Sunderland NHS Foundation Trust, Sunderland, UK; 15 University Hospitals Birmingham NHS Foundation Trust, Birmingham, UK; 16 East and North Hertfordshire NHS Trust, Stevenage, UK; 17 University Hospitals of Leicester NHS Trust, Leicester, UK; 18 North Cumbria University Hospitals NHS Trust, Carlisle, UK; 19 Derby Teaching Hospitals NHS Foundation Trust, Derby, UK; 20 Northampton General Hospital NHS Trust, Northampton, UK; 21 South Tees Hospitals NHS Foundation Trust, Middlesbrough, UK; 22 Nottingham University Hospitals NHS Trust, Nottingham, UK; 23 The Royal Wolverhampton NHS Trust, Wolverhampton, UK; 24 NHS Ayrshire & Arran, Kilmarnock, UK; 25 University College London Hospitals NHS Foundation Trust, London, UK; 26 University College London, Cancer Research UK & UCL Cancer Trials Centre, London, UK

**Keywords:** Oral cancer, Oral cancer

## Abstract

**Background:**

Guidelines remain unclear over whether patients with early stage oral cancer without overt neck disease benefit from upfront elective neck dissection (END), particularly those with the smallest tumours.

**Methods:**

We conducted a randomised trial of patients with stage T1/T2 N0 disease, who had their mouth tumour resected either with or without END. Data were also collected from a concurrent cohort of patients who had their preferred surgery. Endpoints included overall survival (OS) and disease-free survival (DFS). We conducted a meta-analysis of all six randomised trials.

**Results:**

Two hundred fifty randomised and 346 observational cohort patients were studied (27 hospitals). Occult neck disease was found in 19.1% (T1) and 34.7% (T2) patients respectively. Five-year intention-to-treat hazard ratios (HR) were: OS HR = 0.71 (*p* = 0.18), and DFS HR = 0.66 (*p* = 0.04). Corresponding per-protocol results were: OS HR = 0.59 (*p* = 0.054), and DFS HR = 0.56 (*p* = 0.007). END was effective for small tumours. END patients experienced more facial/neck nerve damage; QoL was largely unaffected. The observational cohort supported the randomised findings. The meta-analysis produced HR OS 0.64 and DFS 0.54 (*p* < 0.001).

**Conclusion:**

SEND and the cumulative evidence show that within a generalisable setting oral cancer patients who have an upfront END have a lower risk of death/recurrence, even with small tumours.

**Clinical Trial Registration:**

NIHR UK Clinical Research Network database ID number: UKCRN 2069 (registered on 17/02/2006), ISCRTN number: 65018995, ClinicalTrials.gov Identifier: NCT00571883.

## Background

Oral squamous cell carcinoma (OSCC) is the eleventh most common cancer worldwide,^1^ with 369 000 new cases annually and rising.^1,2^ Patients with early stage disease (T1/T2) and no overt neck disease (N0) are usually treated surgically, but there has been uncertainty over the best management of the neck because of the presence of occult neck metastasis that are clinically and radiologically undetectable.^3,4^ Some surgeons only resect the primary tumour, reserving neck dissection as salvage treatment for subsequent neck metastasis. Others perform elective neck dissection (END) simultaneous with the mouth tumour resection. The long-standing question remains whether delaying neck dissection until neck metastasis is clinically detectable undertreats the third^5,6^ of patients with occult neck disease and prejudices their survival, or whether offering END upfront overtreats the two-thirds without occult neck disease, unnecessarily increasing morbidity and cost.

END requires a longer, more complex and expensive operation. However, those patients having primary cancer resection only who later develop neck metastases have an increased number of positive neck nodes and extra-capsular spread than are found at END.^7^ Therefore salvage usually necessitates more radical neck surgery and additional chemotherapy and radiotherapy resulting in increased morbidity and adverse psychological impact. The extra treatment increases treatment costs and may cause delay or inability for patients to return to work.

Since the 1990s, improved survival rates were largely attributed to using upfront END.^8^ Until 2015 there had been only four randomised trials but they were old (conducted between 1980 and 2009) and small (each 67–75 patients).^9–12^ Only one strongly supported END.^11^ In May 2015 a large trial at the Tata Memorial Hospital, Mumbai (496 patients) with 3 year follow-up reported a clear benefit for END on overall and disease-free survival.^13^ The Mumbai trial^13^ was conducted at a renowned largely tertiary cancer centre treating around 9,000 oral cancers annually, where disease characteristics and patient management may differ from smaller centres and Western populations, so its results may not be readily generalisable across multiple hospitals in a routine surgical cancer setting, particularly those who treat smaller patient numbers.

Despite the Mumbai trial, the US National Comprehensive Cancer Network (NCCN) 2018 guidance continues to state: “*It is debatable whether or not patients with early stage node-negative oral cavity cancers should receive elective neck dissection*”.^14^ Also, although one UK guideline recently included END as a treatment option,^15^ in 2018 the UK National Institute for Healthcare and Clinical Excellence (NICE) considered that the previous five trials (including the Mumbai trial^13^) together provide evidence that is graded as low quality for overall survival and moderate quality for recurrence-free survival, primarily due to three trials being conducted before 2000; concerns over randomisation and allocation concealment in some studies; and only one contemporary trial. Therefore, NICE did not consider that the five trials to date provide sufficient evidence to make stronger recommendations.^16^ Furthermore, out of all five trials before SEND, four were single centre only and the other involved only three hospitals all within Hong Kong.^12^

We report the results of a trial of END (the Selective Neck Dissection study, SEND). This is the only multicentre national randomised study comparing local resection with or without END in early mouth cancer, and in a Western population. The results provide high-quality evidence applicable to any specialist surgeon and hospital, such that national recommendations can be strengthened.

## Methods

### Study design

We conducted a randomised controlled study to determine whether END with local resection improves outcomes in patients with early stage OSCC, compared to those who have resection only. The trial had national ethics approval and all patients gave written informed consent. Potential patients were discussed by a multi-disciplinary team (MDT), and eligible if: aged ≥16 years; had histopathologically confirmed T1/T2 OSCC; no evidence (clinical, including preoperative imaging) of nodal involvement in the neck (N0); and they did not have cancer of the lip, previous oral/pharyngeal SCC or other synchronous tumour. Allocation concealment was achieved using a central randomisation system. Patients were randomised (1:1 allocation) after research nurses telephoned Saving Faces, the trial co-ordinating centre. A minimisation computer program stratified patients by age (<40, 40–64, 65+years), tumour stage (T1 and T2) and surgeon. Patients were staged using the American Joint Committee Cancer (AJCC) 6^th^ edition, then 7^th^ edition after 2010.

### Observational (real world) cohort

As with many surgical randomised trials, eligible patients often decline to participate, or their surgeon may have a preference for one surgical method for a particular individual. A survey of three UK surgical trials in head and neck cancer (including SEND) showed that problems with recruitment were due to patient/surgeon preference, patient aversion to randomisation and insufficient time in clinics for research.^17^ Rather than ignoring these patients we specifically included them in the SEND protocol, to be analysed separately to the randomised group as a form of real-world cohort, to help address potential selection bias amongst the randomised patients. We obtained ethical approval and patient consent to collect data prospectively from the medical records of eligible patients who either declined randomisation or were not approached. These data would be used as supporting evidence for the main trial, acknowledging potential differences in patient characteristics. This approach of examining evidence from both randomised trials and real-world data from routine practice can give a more comprehensive account of the efficacy and safety of a therapy.^18^

### Interventions and procedures

For control patients, resection of the primary tumour was done through the open mouth, without neck incisions or neck surgery. Surgeons aimed for a >5 mm clearance of the tumour at all margins and in all planes. Reconstruction was allowed but not if it involved any neck surgery.

The standard END involved harvesting lymph nodes from levels I–IV including levels Ia/b and IIa/b on the same side as the tumour (ipsilateral). However, a level l–lll dissection and exclusion of level IIb could be performed if considered appropriate. The omohyoid muscle and posterior belly of the digastric muscle could be removed, but the internal jugular vein, sternomastoid muscle and accessory nerves were preserved and left in situ. If the tumour extended to the midline, a neck dissection on the contralateral side was performed with ipsilateral END.

If the cancer recurred in the mouth or neck, surgery (further resection and/or neck dissection) could be done according to local practice, with radiotherapy and/or platinum chemotherapy as recommended locally based on national guidelines.

Patients were examined at baseline (demographics, quality of life-QoL and initial tumour measurement), then two-monthly (years 1 and 2), three-monthly (year 3) and 6-monthly until 5 years. Patients in the observational cohort had the same clinical follow-up schedule as those in the randomised trial.

### Imaging

Baseline imaging scans were mandatory to detect features suggestive of clinically occult neck metastasis and any patients with these features were excluded. Almost all centres used ultrasound with fine needle aspiration cytology (US-FNAC) to detect neck metastasis at the trial outset but some were still using CT or MRI. By the end of the trial US-FNAC was universally used. The standard features of abnormal size and shape, central necrosis (regardless of nodal size) and alteration in vascularity of the lymph nodes were used as radiological markers for metastasis (to exclude patients).

Imaging for suspected locally recurrent, metastatic or new disease was arranged in the event of clinical suspicion or if the patient reported worrying symptoms. This was performed according to local practice. MRI and CT were usually used to detect local disease; US-FNAC and MRI were used to detect neck disease; and positron emission tomography with CT (PET-CT) to detect distant metastases.

### Pathology

Tumour samples from the mouth (all patients) and neck (END patients only), at baseline and recurrence, were sent for local pathological assessment. All specimens were processed and examined using the Royal College of Pathologists minimum dataset for oral cancer. For patients with suspected local or neck recurrence, lesions were usually accessible for pathological sampling and diagnosis, but diagnosis of inaccessible distant metastases was based on radiological findings.

### Outcomes

The primary outcome was overall survival (OS; death from any cause, and those who did not die were censored when last known to be alive). Secondary outcomes included: disease-free survival (DFS: local, regional or distant recurrence, a new primary tumour or death from any cause, whichever occurred first, and patients without an event were censored when last known to be alive); loco-regional recurrence (any recurrence in the mouth from the original tumour, or occurrence/recurrence in the neck, but excluding a new primary mouth tumour and death from oral cancer without a prior diagnosis of recurrence); adverse events (graded as mild, moderate, severe, or life threatening/disabling) occurring up to 6 months post-surgery, which is when surgically-related events would usually occur. Health-related quality of life was assessed using the EORTC-QLQ-C30 and head and neck cancer specific module at baseline and 6 months later.^19^ NHS resource use (number of hospital inpatient days, outpatient days, and GP visits) was collected up to 24 months after surgery, using a questionnaire completed by patients when they attended the clinic for follow-up (every 2 months).

### Statistical considerations

We aimed for a 10 percentage point improvement with END, from a 5-year OS rate of 65% using resection alone. This required 620 patients, with 80% power and two-sided 5% statistical significance.

Time-to-event outcomes were measured from the date of surgery or excision biopsy (if it had removed the tumour) because it was the only standard and comparable date. Although time-to-event endpoints are usually measured from the randomisation date, in our trial the surgery date sometimes occurred a few weeks after randomisation and in 22 patients the diagnostic biopsy (wide-local excision with clear margin) had completely excised the mouth tumour, so the surgery date was effectively the biopsy procedure date in these cases. Time-to-event outcomes were analysed using Cox regression (hazard ratio, HR), after confirming the assumption of proportional hazards. The OS HR was also obtained after allowing for the randomisation stratification factors (used as strata in the Cox regression). The worst grade of adverse event for each patient and each type was compared using Fisher’s test. Primary analyses were by intention-to-treat (ITT). Pre-specified per-protocol analyses were performed for patients who had the randomised surgical procedure.

In July 2015, the Independent Data Monitoring Committee (IDMC) recommended early termination of accrual because the preliminary data were consistent with the published Mumbai trial, which showed a benefit for END.^14^ They considered that continued randomisation would be difficult. We collected outcome data for another three years (up to August 2018) to complete about 5 years follow-up, fully capture events and determine long-term outcomes not captured in any previous trial.

### Meta-analysis

All prior randomised trials of END for early stage oral cancer with N0 disease are already known, because they were included in a systematic review in 2015.^20^ We used the same selection criteria (within PUBMED) to confirm that there are no other trials since then, except ours. We performed a meta-analysis of all six trials to produce the complete evidence base, which has not been done before. Inclusion of the two high-quality trials (Mumbai and SEND) should allow the accumulated evidence to be given a higher grading in national guidelines. Key summary patient characteristics were extracted from the five trial publications (including age, stage and differentiation), in addition to the HR for OS and DFS. RevMan software was used to pool the results using the method by Dersimonian and Laird that allows for heterogeneity.^21^

## Results

Seven hundred seventy-six patients with T1/T2 tumours and N0 disease were screened for eligibility. Six hundred fourteen consented to participate in either the randomised study or observational cohort (CONSORT diagram in Supplementary Fig. 1). These 614 patients were operated on by 68 surgeons from 27 hospitals across the UK National Cancer Research Network. Two hundred fifty-five patients agreed to be randomised and they came from 25 hospitals (18 June 2007 to 10 July 2015), and were operated on by 52 surgeons; five patients were found to be ineligible and so excluded from all analyses. Median follow-up was 57 months (25–75^th^ centile 43–61 months). Similar numbers of patients in both trial groups attended each assessment visit.

Among the 22 patients whose tumour had been removed by the diagnostic biopsy, 15 had been randomised to have resection only, of which eight did have the planned resection procedure afterwards but this sample showed no evidence of malignancy, while seven patients had no further procedures after the diagnostic biopsy. Seven of the 22 patients had been randomised to END, and five of these had a neck dissection and two did not (so these two effectively had a resection only).

In those randomised to have resection only, seven patients actually had a neck dissection as well and for another patient what they had was unreported. In those allocated to END, 12 had resection only. Excluding these 20 patients formed the per-protocol group. Almost all neck dissections were performed as one-stage procedures (simultaneous with local resection), three cases had two-stage.

Patient characteristics (Table 1) and tumour characteristics (Supplementary Table 1) were balanced within the randomised patients. Eighty percent (199/250) occurred on the tongue or mouth floor, and 68% (170/250) and 21% (53/250) were staged as pT1 and pT2, respectively. Among patients who were randomised to and received END, 25.4% (29/114) had positive neck nodes (occult neck metastases): 19.1% and 34.8% among those with clinical stage T1 or T2, respectively); or 20.8% and 36.0% among those with pT1 or pT2.

**Table 1:** Baseline patient and tumour characteristics

	SEND (randomised patients)		SEND (observational cohort)	
	Resection only *N* = 124	Neck dissection *N* = 126	Resection only *N* = 234	Neck dissection *N* = 112
Age, median (range), years	63 (31–89)	62 (34–94)	67 (22–95)	61 (28–91)
Gender				
Female	45 (36.3)	44 (34.9)	118 (50.4)	41 (36.6)
Male	79 (63.7)	82 (65.1)	110 (47.1)	70 (62.5)
Unknown			6 (2.5)	1 (0.9)
T-stage				
T1	80 (64.5)	79 (62.7)	138 (59.0)	29 (25.9)
T2	44 (35.5)	47 (37.3)	48 (20.5)	71 (63.4)
Unknown			48 (20.5)	12 (10.7)
Ethnic origin				
Caucasian	106 (85.5)	107 (84.9)	198 (84.6)	99 (88.4)
South East Asian	8 (6.4)	14 (11.1)	19 (8.1)	7 (6.2)
African/Caribbean	3 (2.4)	1 (0.8)	1 (0.4)	
Other/unknown	7 (5.7)	4 (3.2)	16 (6.8)	6 (5.4)
Smoking status				
Never	33 (26.6)	44 (34.9)	75 (32.0)	30 (26.7)
Former	45 (36.3)	41 (32.5)	106 (45.3)	46 (41.1)
Current	45 (36.3)	41 (32.5)	45 (19.2)	33 (29.5)
Unknown	1 (0.8)		8 (3.4)	3 (2.7)
Site of primary tumour				
Tongue	70 (56.5)	82 (65.1)	152 (65.0)	75 (67.0)
Floor of mouth	23 (18.5)	24 (19.0)	29 (12.4)	25 (22.3)
Buccal mucosa	16 (12.9)	8 (6.4)	20 (8.6)	6 (5.4)
Gingivae	10 (8.1)	5 (4.0)	11 (4.7)	
Palate	1 (0.8)	1 (0.8)	9 (3.8)	2 (1.8)
Tonsil		2 (1.6)	2 (0.8)	
Two or more of the above	3 (2.4)	4 (3.2)	10 (4.3)	4 (3.6)
Unknown	1 (0.8)		1 (0.4)	
Differentiation				
Well	17 (13.7)	16 (12.7)	43 (18.4)	12 (10.7)
Moderate	73 (58.9)	70 (55.6)	130 (55.6)	61 (54.5)
Poor	22 (17.7)	30 (23.8)	36 (15.4)	34 (30.4)
Unknown/not assessable	12 (9.7)	10 (7.9)	25 (10.7)	5 (4.5)
pN-stage				
N0	5 (4.0)^a^	85 (67.5)		68 (60.7)
N1	2 (1.6)^a^	20 (15.9)		21 (18.7)
N2 or N3	–	9 (7.1)		17 (15.2)
NX/unknown	117 (94.4)	12 (9.5)^b^		6 (5.4)
Median (range), mm				
Maximum tumour diameter^c^	15 (1–39)	15 (2–40)	14 (1.5–40)	22 (3–50)
Maximum tumour diameter^d^	12.5 (1–40)	14 (0.5–44)	12 (1–47)	18 (1–45)
Maximum depth of invasion^d^	4.5 (0.3–19)	5 (0.2–29.4)	4 (0.7–19)	7.5 (1–25)

^a^All 7 had a neck dissection

^b^All 12 had resection only

^c^Estimated by the surgeon

^d^Pathology assessment

### Efficacy (randomised patients)

**Fig. 1:**
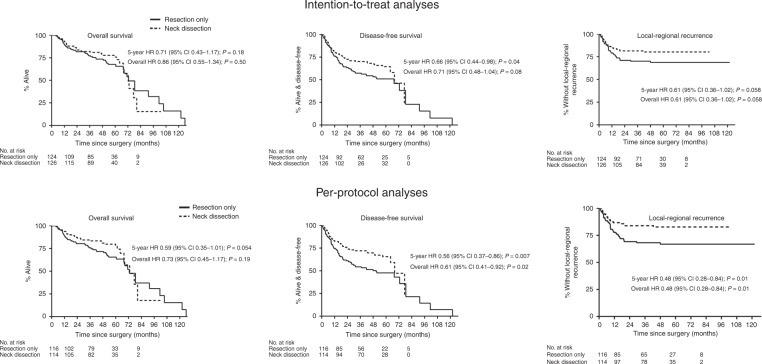
Measures of efficacy for the randomised patients. The 5-year OS HR allowing for the randomisation stratification factors (age, T-stage and surgeon; stratified intention-to-treat analysis) is 0.64 (95% CI 0.33–1.23). Because 41 surgeons each operated on <10 patients, we also replaced ‘surgeon’ with geographical location and the adjusted OS is 0.73 (95% CI 0.44–1.22). All three efficacy outcomes (OS, DFS, loco-regional recurrence) did not violate the assumption of proportional hazards (the OS curves crossed when there were few patients). (OS overall survival, DFS disease-free survival, HR hazard ratio, CI confidence interval)

Summary efficacy results are shown in Table 2 and Fig. 1. Among the 250 patients, there were 83 deaths (49 due to oral cancer). From the ITT analysis, the 5-year OS rates were 75.8% END versus 67.6% resection only: difference of 8.2 percentage points (*p* = 0.28). The 5-year hazard ratio (HR) was 0.71 (*p* = 0.18); Fig. 1.

**Table 2:** Summary efficacy results according to intention-to-treat and per-protocol analyses (the latter excludes patients who did not have the surgical procedure they were randomly allocated to)

	Total no. of events	5-year absolute risk difference (95% CI) END minus resection only	Hazard ratio: up to 5 years (95% CI)	Hazard ratio: all time points (95% CI)
Intention-to-treat (250 patients)				
Overall survival	83	8.2 (−6.7, 23.1) *p* = 0.28	0.71 (0.43–1.17) *p* = 0.18	0.86 (0.55–1.34) *p* = 0.50
Disease-free survival	109	13.5 (−2.0, 29.0) *p* = 0.087	0.66 (0.44–0.98)^a^ *p* = 0.04	0.71 (0.48–1.04)^a^ *p* = 0.08
Loco-regional recurrence	60	11.5 (−3.5, 26.5) *p* = 0.13	0.61 (0.36–1.02) *p* = 0.058	0.61 (0.36–1.02) *p* = 0.058
Per-protocol (230 patients)				
Overall survival	77	11.9 (−3.8, 27.6) *p* = 0.14	0.59 (0.35–1.01) *p* = 0.054	0.73 (0.45–1.17) *p* = 0.19
Disease-free survival	103	17.9 (1.7, 34.0) *p* = 0.03	0.56 (0.37–0.86)^a^ *p* = 0.007	0.61 (0.41–0.92)^a^ *p* = 0.02
Loco-regional recurrence	55	15.8 (0.3, 31.3) *p* = 0.045	0.48 (0.28–0.84) *p* = 0.01	0.48 (0.28–0.84) *p* = 0.01

*END* elective neck dissection with mouth resection

^a^Excluding two patients who had cancer of the tonsil and one patient with unknown site, the DFS hazard ratios became 0.64 (up to 5 years) and 0.69 (all time points) for intention-to-treat analyses; and 0.54 and 0.59 for the corresponding per-protocol analyses

The influence of non-adherence was noticeable in this study size. Seven patients allocated to resection only but who actually had an END were all alive and disease-free at last follow-up but of 12 patients allocated to END who instead had resection only, five had a cancer occurrence in the neck (all within 13 months from surgery), and one had a new primary mouth tumour; all six died. These observations are better captured in the per-protocol analyses and provide additional favourable evidence for END. The 5-year OS rates based on the per-protocol groups were 77.5% END versus 65.6 resection only (230 patients and 77 deaths): risk difference 11.9 percentage points (*p* = 0.14). The corresponding HR up to 5 years was 0.59 (*p* = 0.054); Fig. 1.

There were 109 DFS events (Supplementary Table 2). The ITT 5-year DFS rate was 64.4% END versus 50.9% resection only (risk difference 13.5 percentage points, *p* = 0.087), and 65.7% versus 47.8% using the per-protocol analysis (risk difference 17.9 percentage points, *p* = 0.03). The 5-year DFS HRs were statistically significant. Similar benefits were seen for loco-regional occurrence/recurrence in the mouth or neck.

Almost all 60 loco-regional disease occurrence/recurrence DFS events were seen within two years of surgery, and only three (1 END, 2 resection only) occurred after this time (Fig. 1). Six patients (three in each group) developed new primary mouth tumours more than 2 years after surgery.

Pre-specified subgroup analyses (per-protocol) were used to show that the effect of END was not substantially different between subgroups (Supplementary Figs. 2, 3). Importantly, END improved clinical outcomes among small tumours. The DFS HRs were 0.38 (95% CI 0.21–0.70) for T1 clinical stage, 0.49 (95% CI 0.28–0.86) for pathology T1 stage, 0.63 (95% CI 0.27–1.49) for pathology-assessed tumour diameter ≤10 mm, and 0.64 (95% CI 0.24–1.73) for patients with both clinical stage T1 and diameter ≤10 mm (Supplementary Fig. 3).

A recent study suggested that END may be unnecessary for well-differentiated T1-stage tumours,^22^ but in SEND the DFS HR for this subgroup was 0.54 (95% CI 0.13–2.16). We also conducted analyses (not pre-specified) according to the AJCC 8th edition,^23^ because it uses depth of invasion and tumour size. The per-protocol OS HRs were 0.76 (95% CI 0.34–1.71), 0.64 (95% CI 0.25–1.67) and 0.73 (95%  CI 0.26–2.06) for stage T1, T2 and T3, respectively, with corresponding DFS HRs 0.51 (95% CI 0.28–0.93), 0.75 (95% CI 0.35–1.60) and 0.75 (95% CI 0.30–1.85); confirming that even patients with favourable staging still benefit from END.

### Adverse events

Table 3 summarises the adverse events (details in Supplementary Tables 3, 4). Although more patients had an adverse event of any grade in the END group 77.8% END (98/126) versus 60.5% resection only (75/124) *p* = 0.003, the majority were low-grade events. There was no major difference for grade 3–4 events: 19.8% END (25/126) versus 14.5% resection only (18/124), *p* = 0.26. Neck sensory and motor nerve abnormalities, and problems with swallowing were more common with END. Among patients with T1 tumours (Supplementary Table 5), the percentage with any grade of event was 77.2% (61/79) END versus 62.5% (50/80) resection only, *p* = 0.04; and corresponding values 17.7% (14/79) versus 12.5% (10/80), *p* = 0.36, for grade 3–4 events, similar for all patients.

**Table 3:** Adverse events among randomised patients occurring up to 6 months from the baseline surgery (based on the maximum grade for each patient and each event type); the number of patients for whom the event was still ongoing at 6 months is shown in square brackets

	Resection only *N* = 124 (%)		Neck dissection and resection *N* = 126 (%)		*P*-value (Fisher’s exact test)
	Grade 1–2/unknown	Grade 3–4	Grade 1–2/unknown	Grade 3–4	
Nerve damage (face or neck)	17 (13.7) [10]	1 (0.8) [1]	44 (34.9) [29]	6 (4.8) [3]	*P* < 0.001
Problems in mouth	26 (20.9) [11]	2 (1.6) [1]	28 (22.2) [11]	3 (2.4) [1]	*P* = 0.92
Problems swallowing	8 (6.4) [1]	2 (1.6)	19 (15.1) [5]	6 (4.8)	*P* = 0.03
Speech/vocal cord problems	7 (5.6) [4]	2 (1.6) [1]	9 (7.1) [5]		*p* = 0.49
Swollen glands/swelling in mouth or neck	18 (14.5) [7]	2 (1.6)	15 (11.9) [2]	4 (3.2) [2]	*P* = 0.63
Problems taste/hearing	1 (0.8) [1]		1 (0.8) [1]	3 (2.4) [1]	*P* *=* 0.75
Wound healing problems	10 (8.1) [2]	2 (1.6)	14 (11.1)	3 (2.4)	*P* = 0.66
Possibly related to chemo/RT^a^	19 (15.3)	3 (2.4)	22 (17.5)	2 (1.6)	*P* = 0.83
Any event recorded (each patient counted once)	57 (46.0)	18 (14.5)	73 (57.9)	25 (19.8)	*P* = 0.01

All events were grade 3, except the following were grade 4:

Resection only: one patient with accessory nerve damage, one with problems swallowing, one with swollen glands/swelling in mouth/neck.

Neck dissection: two who had a tracheostomy (within ‘problems swallowing’), one with swollen glands/swelling in mouth or neck.

^a^Weight loss, diarrhoea, nausea/vomiting, skin rash, abnormal biochemistry, dry mouth and limited mouth opening after radiotherapy

### Further interventions

Supplementary Table 6 shows additional surgery and use of chemotherapy/radiotherapy, including when they were given following disease recurrence (salvage). Among the 29 patients who had an END in which N1/N2 disease was found (Table 1), 13 were known to have received adjuvant radiotherapy or platinum chemo-radiotherapy. Further neck dissections during follow-up were known to have been performed in 12.7% (16/126) patients who initially had an END, compared to 19.4% (24/124) who initially had resection only (*p* = 0.15). As anticipated (see Introduction), nearly twice as many patients who had resection only had chemo-radiotherapy after a recurrence: 19.4% (24/124) versus 10.3% (13/126) using END, *p* = 0.04.

### Outcomes in the observational cohort

Many characteristics were similar between the randomised and observational cohorts; Table 1 and Supplementary Table 1. The different proportions for T-stage and tumour diameter were expected because surgeons are more likely to recommend an END for large tumours, and resection only for smaller tumours (outwith a randomised trial). Among those who had an END, 33.9% had positive neck nodes (17.2% and 40.8% in clinical stage T1 or T2 patients, respectively).

**Fig. 2:**
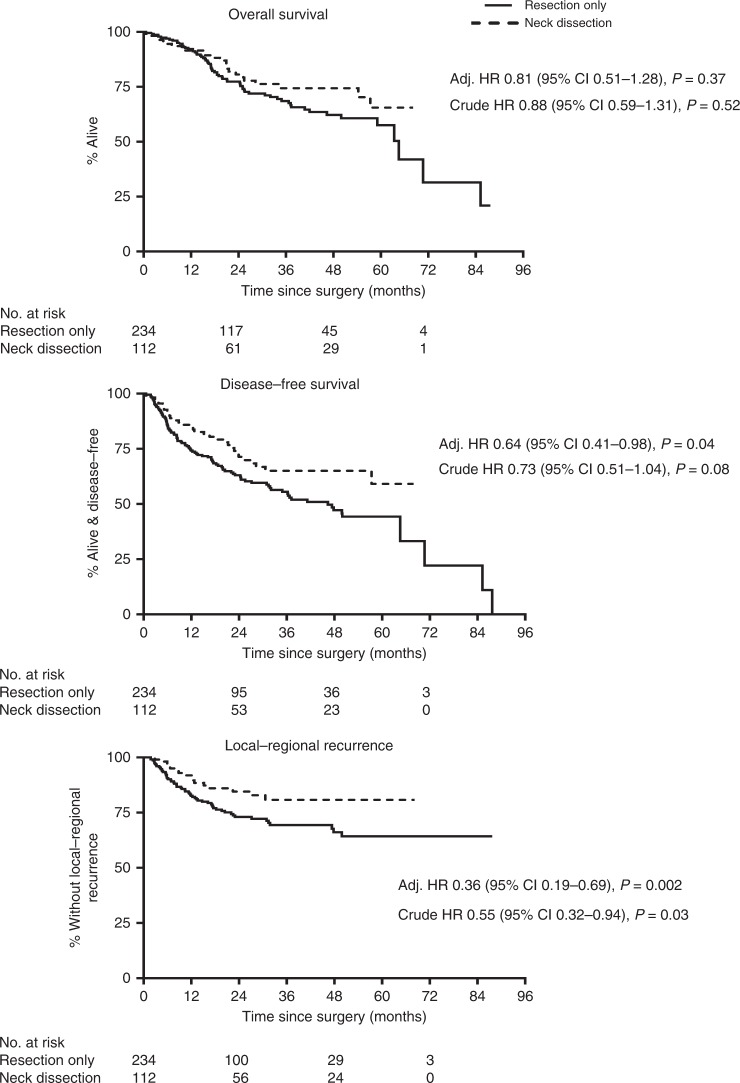
Patients in the observational cohort. The adjusted hazard ratios allow for age, sex, smoking status, alcohol drinking status, geographical location, clinical T-stage and site of tumour in the mouth. If additionally adjusted for tumour pathology features (tumour diameter, depth of invasion, differentiation and completeness of resection), the HRs become 0.43 (95% CI 0.25–0.75, *p* = 0.003) for OS; 0.35 (95% CI 0.25–0.69, *p* < 0.001) for DFS and 0.19 (95% CI 0.09–0.44, *p* < 0.001) for loco-regional recurrence. (OS overall survival, DFS disease-free survival, HR hazard ratio, CI confidence interval)

Two hundred thirty-four patients had resection only and 112 had an END, of which 113 and 41, respectively, had a first event (recurrence/died; Supplementary Table 2). Figure 2 shows that END was associated with better outcomes than resection only, with HRs adjusted for patient characteristics: HR = 0.81 (*p* = 0.37), 0.64 (*p* = 0.04) and 0.36 (*p* = 0.002) for OS, DFS and loco-regional recurrence, respectively. These effects were larger when pathological features of the mouth tumour were also accounted for: HR = 0.43 (*p* = 0.003), 0.35 (*p* < 0.001) and 0.19 (*p* < 0.001), and the adjusted Kaplan–Meier curves for this are shown in Supplementary Fig. 4.

The benefits for END were also seen among patients with small tumours. Adjusted HRs for pT1-stage tumours were 0.58 (*p* = 0.17) for OS, HR = 0.48 (*p* = 0.03) for DFS and HR = 0.33 (*p* = 0.02) for loco-regional recurrence.

Although this patient cohort cannot be used on its own to reliably estimate the magnitude of the benefit for END, it supports the randomised trial findings. Figure 2 (like Fig. 1) shows that loco-regional recurrences are uncommon after 2 years post-surgery.

Among the 38 patients who had an END in which N1/N2 disease was found (Table 1), 20 were known to have received adjuvant radiotherapy or platinum chemo-radiotherapy. Details of further treatments given are shown in Supplementary Table 6, including salvage therapies following recurrence. Further neck dissections, with or without a mouth tumour resection, were seen in 8.0% (9/112) patients who initially had an END, but 25.6% (60/234) who initially had resection only (*p* < 0.001). A further mouth tumour resection (no neck dissection at all) was seen in 3.6% (4/112) and 14.1% (33/234) among patients who initially had END or resection only, respectively. More patients had surgery for complications among those who received END 12.5% (14/112), compared to resection only 3.0% (7/234).

Supplementary Table 7 summarises the adverse events, which were similar to the randomised trial (Table 3). The proportion with grade 3 events following an END was low (12.5% patients).

### Meta-analysis of all randomised trials

**Fig. 3:**
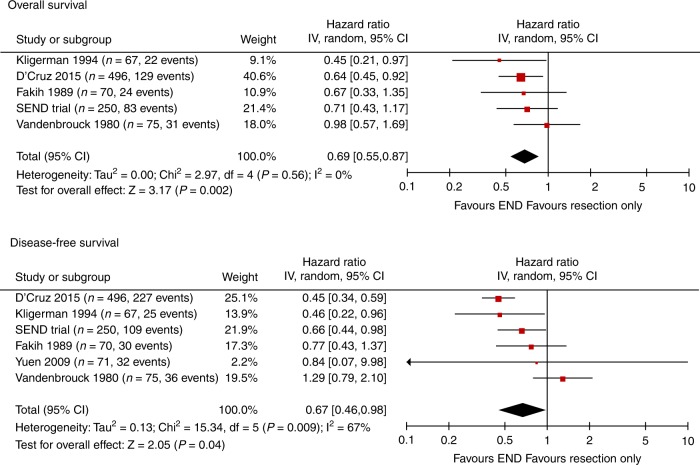
Forest plots of all randomised trials^9–13^ that have evaluated elective neck dissection (END) for early stage oral cancer. All trials except one compared END with resection only of the primary mouth tumour, whilst in the study by Vandenbrouck^9^ all patients had radiotherapy for the primary tumour and were then randomised to receive a neck dissection or not. Excluding the Vandenbrouck study produces *p* = 0.35 for the heterogeneity test and I^2^ = 11% for DFS, and the pooled HR is 0.54, 95% CI 0.43–0.68, *p* < 0.001 for DFS, and HR 0.64, 95% CI 0.49–0.82 *p* < 0.001 for OS. (OS overall survival, DFS disease-free survival, END elective neck dissection, HR hazard ratio, CI confidence interval)

There are now six randomised studies of END in early stage cancer that have ever been conducted. All trials except one compared END with resection only, whilst in this one trial^9^ all patients had radiotherapy for the primary mouth tumour instead of surgery (Supplementary Table 8). Figure 3 shows forest plots for OS and DFS. The pooled HR for OS indicates a 31% reduction in the risk of death with END (HR = 0.69, *p* = 0.002) and 33% reduction in the risk of recurrence/death (DFS HR = 0.67, *p* = 0.04).

The apparent heterogeneity for disease-free survival was due to the trial using radiotherapy to treat the primary^9^ which if excluded produced *p* = 0.35 for the heterogeneity test and I^2^ = 11%, and pooled HR 0.54 (*p* < 0.001) for DFS, and OS HR 0.64 (*p* < 0.001). These pooled HRs are more appropriate because this trial^9^ had a different background therapy to the others and 13% of patients had T3 tumours.

When combining only the two modern high-quality trials (SEND and the Mumbai study),^13^ the pooled HR for OS was 0.66 (95% CI 0.49–0.89, *p* = 0.006), and for DFS 0.53 (95% CI 0.37–0.77, *p* < 0.001), both clearly in favour of END.

### Health-related quality of life (randomised patients)

Supplementary Figs 5 and 6 show the change in scores from baseline to 6 months post-surgery. Patients who had an END had very similar QoL scores to those who had resection only for many QoL measures, including emotional functioning and problems swallowing. Statistically significant differences were only seen for dry mouth (END patients had worsening QoL, *p* = 0.03) and nausea (END patients had better QoL, *p* = 0.02). There were suggestions that END patients had worsening social contact issues (*p* = 0.07) and problems with work/daily activities (*p* = 0.07).

### Resource use

Patients who received an END spent a median of 6 days in hospital (10–90th centile 4–14 days) for the procedure, compared to 2 days for those who had a resection only (10–90th centile 1–7 days); Wilcoxon *p* < 0.001. Up to 2 years after the initial operation, the median number of inpatient days was 4.5 with END (10–90th centile 1–35) versus 3 with resection only (10–90th centile 0–12), *p* = 0.13. There was also no material difference in either the number of outpatient visits: median 6 visits in the END patients (10–90th centile 1–28) versus 7 in the resection only group (10–90th centile 2–28), *p* = 0.46; or number of GP visits, with median 4 in each group (10–90th centile 1–11 for END and 1–13 for resection only), *p* = 0.85.

### Features of neck disease (randomised patients only)

Disease that later occurs in the neck is of interest when considering the role of END. Supplementary Table 9 compares features of patients who had a neck recurrence after END (*n* = 14) or neck metastasis after resection only (*n* = 37), with those who remained disease-free and alive during the trial. At baseline, the resection-only patients who subsequently developed neck metastasis were more likely to have poorly differentiated tumours, stage pT2, discohesive invasive front, tumour margins <5 mm, perineural/vascular/lymphatic invasion and larger tumour diameter and depth of invasion compared to patients who were alive and disease-free but also had resection only. Similar observations were seen for those who had an END and developed a subsequent neck recurrence. Neck node necrosis and extra-capsular spread were more common among patients who had larger primary tumour diameters and were also more commonly found in resection only patients who subsequently had a neck dissection for neck metastasis or in END patients who developed a neck recurrence after END.

## Discussion

Although recruitment stopped early, the results from SEND demonstrate that END improved OS and DFS, and reduced loco-regional recurrence, though only the last two endpoints were statistically significant. The observed improvement with END (8.2 percentage points with ITT and 11.9 with per-protocol analyses) are close to our target (10 percentage points). The results from the randomised patients were supported by those from the prospective cohort from the same centres. All the evidence taken together (our multicentre national randomised trial plus a real-world cohort from the same centres, and meta-analysis of all randomised studies) provides high-level quantifiable evidence for the survival benefit of END. END is associated with an increased risk of low-grade adverse events including effects on neck motor and sensory nerves and increased length of hospital stay. END had minimal impact on most QoL components.

The SEND trial complements the 2015 Mumbai trial^13^ because of the differences in patient characteristics, clinical outcomes and lengthier follow-up. The observed large treatment effect for END (DFS HR 0.45) in the Mumbai trial might be partly due to it coming from an internationally renowned institution, and with relatively few surgeons involved in the trial, most of whom were trained “in house”. Therefore, its results may not be generalisable to every head and neck cancer surgeon. Conversely, the SEND trial generated results with surgeons who trained and operate in many different institutions so are more representative of all head and neck cancer centres and surgeons (DFS HR 0.66).

Several patient factors differed between the Mumbai^13^ and SEND trials: the mean ages 48 (Mumbai) versus 63 years (SEND); male 75% versus 64%; tongue tumours 85% versus 61%; floor of mouth 1% versus 19%; and T1-stage 44% versus 64%. Outcomes also differed between these two trials, with higher survival for resection only patients in SEND. For the Mumbai trial the 3-year DFS rates for END versus resection only were 70% versus 46%, and OS rates 80% versus 67%; while in SEND they were 70% versus 57% (DFS) and 82% versus 77% (OS); consistent with outcomes from recently reported large scale retrospective studies.^24,25^ The higher OS and DFS rates in SEND patients who had resection only could be due to having more favourable disease (lower stage disease) than in the Mumbai patients. Despite these differences, both trials show an improvement in OS (HRs 0.64 and 0.71) and DFS (HRs 0.45 and 0.66), though the effects were more conservative in the UK trial.

Our findings for occult disease of 25.4% in patients who received END as randomised and later neck metastases developing in 27.6% of patients who had resection only as randomised (the corresponding findings for the Mumbai study were 29.6% and 45%) more closely matches findings of 21% where ultrasound with fine needle aspiration cytology is used to determine N0 status for T1/2 tumours.^26^

Many surgeons consider that END is not needed for small mouth tumours. Recent observational studies recommend END but not for patients who have T1-stage with tumour thickness <4 mm,^27^ or those with tumour diameter <10 mm.^28^ However, we showed that END was beneficial for small tumours (pT1 stage) or tumour diameter ≤10 mm, with corresponding DFS HRs of 0.49 and 0.63 respectively (the same conclusions came from our observational cohort). This is an important finding for surgeons because of the uncertainty over the value of END in smaller tumours, which was also implied in the NCCN guidelines.^14^ In fact many surgeons have been under the impression that occult neck metastases in patients with clinical stage T1 tumours is uncommon, but our national study shows that the figure is higher than expected: 19.1% in the randomised trial and 17.2% in the ‘real world’ cohort. In the Mumbai trial, the OS HRs were ~0.75 and 0.42 for T1 and T2 tumours, respectively (suggesting END might be more favourable in the larger tumours), but the difference was not statistically significant. Therefore, both trials together provide evidence for the benefit for END regardless of tumour size. Furthermore, evidence from our randomised study that END also benefits pT1-stage tumours that are well-differentiated, contrasts with the conclusion of a recent retrospective study.^22^

The authors of the Mumbai study indicated that END might not be effective in patients whose tumour has a depth of invasion ≤3 mm (based only on 10 deaths). In our SEND trial, the hazard ratio for overall survival was 1.02 (based on 24 deaths). However, none of the tests for interaction were statistically significant, and it is important to note that the 95% confidence interval for this subgroup included the overall hazard ratio (in each trial), which does not provide evidence for a subgroup effect. Furthermore, in SEND, the hazard ratio for DFS among patients with a depth of invasion ≤3 mm was 0.81 (Supplementary Fig. 3), which is suggestive of a benefit for END.

Adverse events were as expected, consistent with other studies,^13^ including a retrospective analysis (~21,000 patients) showing very low 30-day mortality and re-admission rates for patients with stage T1/T2 tumours.^29^ Health economic analyses show that END is more cost-effective than resection alone with a lifetime cost saving of $6,000 compared to using resection only.^30^

Our randomised study is the only national multicentre randomised trial of END, conducted across 25 centres with multiple radiologists, pathologists and (52) surgeons. The findings are therefore applicable to any head and neck cancer unit. Key strengths of our study include: high-quality data collection from an established national cancer research network; all patients assessed by an MDT; and supporting prospective evidence from concurrent patients in a real-world cohort who had their preferred surgical procedure. We also have the longest follow-up (57 months, compared to 39 months for the Mumbai trial). Furthermore, the SEND study is the only randomised trial of END that collected patient reported outcomes and resource use.

The main limitation is that SEND stopped recruiting early. However, the trial did have sufficient power to show statistically significant improvements for two efficacy endpoints, DFS and loco-regional recurrence, and DFS is now commonly used as the primary outcome measure for early stage cancer treatment trials. Although the OS results were not statistically significant, the HR point estimates were in the direction of benefit.

An important observation from SEND is that in this geographically diverse study, the vast majority (95%) of recurrences or occurrences of cancer in the mouth or neck were seen within 2 years from surgery, as seen by other investigators.^31^ This could have implications for long-term clinical follow-up, in that most patients may not need annual assessments, and could instead be discharged to their GP earlier, particularly if they have no clear adverse prognostic factors.

Sentinel lymph node biopsy (SLNB) is suggested as a surgical alternative to END. It is a reliable technique for staging T1/2 N0 disease,^32^ and considered more accurate than US-FNAC at detecting neck metastases^33^ though more invasive, morbid and costly. SLNB is less invasive than END with reduced morbidity,^34,35^ but SLNB is only diagnostic while END is therapeutic and subsequently needed anyway after a positive SLNB. A small observational study suggested that SLNB was less costly than END,^36^ but no prospective randomised trial has directly compared them. SLNB is resource intensive for histopathology and operating time. Also, patients with false negative results undergo salvage neck dissection later with negative survival results (as with resection only). Few US centres have sufficient expertise to use SLNB,^14^ therefore it is not commonly used there,^35^ and the increased resources needed preclude its use in low/middle income countries. In the few UK centres that use SLNB, some limit its use to thin oral cancers and diameters <5 mms because of the low risk of positive sentinel nodes.

Following the Mumbai trial,^13^ several surgeons consider END on a case-by-case basis, but do not usually use END for T1 tumours. There is also still variability of uptake internationally, probably because NCCN and NICE do not yet recommend END for early stage node-negative oral cancer.^16,17^

The combined evidence presented in this paper including the two largest and contemporary randomised trials (one national multicentre study [SEND] and one from a single tertiary centre), together with real-world data and our meta-analysis now unequivocally demonstrates the survival benefit of END in early stage mouth cancer, including in small tumours.

Our paper should eliminate the uncertainty over END indicated in national guidelines and these should be updated to reflect the benefit from END. The paper also presents quantifiable evidence comparing QoL, resource use and treatment complication rates between the patients treated with and without END. All this information will enable surgeons for the first time to provide clear evidence regarding the benefits and impact of END for their patients thereby enabling patients to participate more effectively with decision-making regarding their treatment.

## Supplementary information

Supplementary Online Tables and Figures

## Data Availability

Data and materials could be made available to those requesting it through a formal collaboration, after a review of the proposal. Please contact Saving Faces—send@savingfaces.co.uk
