# Correction to: Nationwide randomised trial evaluating elective neck dissection for early stage oral cancer (SEND study) with meta-analysis and concurrent real-world cohort

**DOI:** 10.1038/s41416-021-01678-2

**Published:** 2021-12-23

**Authors:** Iain L. Hutchison, Fran Ridout, Sharon M. Y. Cheung, Neil Shah, Peter Hardee, Christian Surwald, Janavikulam Thiruchelvam, Leo Cheng, Tim K. Mellor, Peter A. Brennan, Andrew J. Baldwin, Richard J. Shaw, Wayne Halfpenny, Martin Danford, Simon Whitley, Graham Smith, Malcolm W. Bailey, Bob Woodwards, Manu Patel, Joseph McManners, Chi-Hwa Chan, Andrew Burns, Prav Praveen, Andrew C. Camilleri, Chris Avery, Graham Putnam, Keith Jones, Keith Webster, William P. Smith, Colin Edge, Iain McVicar, Nick Grew, Stuart Hislop, Nicholas Kalavrezos, Ian C. Martin, Allan Hackshaw

**Affiliations:** 1 Barts Health NHS Trust, London, UK; 2 Saving Faces—The Facial Surgery Research Foundation, London, UK; 3 Barking, Havering and Redbridge University Hospitals NHS Trust, Romford, UK; 4 Brighton and Sussex University Hospitals NHS Trust, Brighton, UK; 5 Royal Free London NHS Foundation Trust, London, UK; 6 Portsmouth Hospitals NHS Trust, Portsmouth, UK; 7 The Pennine Acute Hospitals NHS Trust, England, UK; 8 Aintree University Hospital NHS Foundation Trust, Liverpool, UK; 9 Royal Surrey County Hospital NHS Foundation Trust, Guildford, UK; 10 St George’s University Hospitals NHS Foundation Trust, London, UK; 11 University Hospital of South Manchester NHS Foundation Trust, Manchester, UK; 12 NHS Forth Valley, Stirling, UK; 13 Luton and Dunstable Hospital NHS Foundation Trust, Luton, UK; 14 City Hospitals Sunderland NHS Foundation Trust, Sunderland, UK; 15 University Hospitals Birmingham NHS Foundation Trust, Birmingham, UK; 16 East and North Hertfordshire NHS Trust, Stevenage, UK; 17 University Hospitals of Leicester NHS Trust, Leicester, UK; 18 North Cumbria University Hospitals NHS Trust, Carlisle, UK; 19 Derby Teaching Hospitals NHS Foundation Trust, Derby, UK; 20 Northampton General Hospital NHS Trust, Northampton, UK; 21 South Tees Hospitals NHS Foundation Trust, Middlesbrough, UK; 22 Nottingham University Hospitals NHS Trust, Nottingham, UK; 23 The Royal Wolverhampton NHS Trust, Wolverhampton, UK; 24 NHS Ayrshire & Arran, Kilmarnock, UK; 25 University College London Hospitals NHS Foundation Trust, London, UK; 26 University College London, Cancer Research UK & UCL Cancer Trials Centre, London, UK

**Keywords:** Oral cancer, Oral cancer

Correction to: *British Journal of Cancer* **121**:827–836, 10.1038/s41416-019-0587-2, published online 15 October 2019

The original version of this article unfortunately contained a mistake. A correction is needed for one of the trials in the meta-analysis (Fig. 3). For Fakih et al. 1989, the relative risk of death (used as an estimate of the overall survival hazard ratio; as the authors did with other trials that did not report hazard ratios directly) was calculated using 8 deaths among 30 patients who had neck dissection versus 16 deaths among 40 patients who had resection only (RR = 0.67). The authors have since noted that there were 9 deaths among the 30 patients, yielding RR = 0.75 95% CI: 0.39–1.46. This trial had a small weight in the meta-analysis, so the corrected pooled hazard ratio across all 5 trials is now RR = 0.70 (95% CI: 0.55–0.88); almost the same as that published RR = 0.69 (95% CI: 0.55–0.87). Nevertheless, the main focus should be on the authors SEND trial and D’Cruz et al. 2015, as they were the most contemporary and highest quality.

