# Supplementary material for: Nationwide randomised trial evaluating elective neck dissection for early stage oral cancer (SEND study) with meta-analysis and concurrent real-world cohort
Source: Br J Cancer. 2019 Oct 15;121(10):827–36. doi: 10.1038/s41416-019-0587-2 (PMC6888839; doi:10.1038/s41416-019-0587-2)
Supplement: Supplementary file 1 — Supplementary Online Tables and Figures [file 41416_2019_587_MOESM1_ESM.docx]

**Supplementary Online Tables and Figures**

We are grateful to all the surgeons involved in recruiting patients and performing the operations (listed in alphabetical order by centre name):

- Aintree University Hospital NHS Foundation Trust: Fazilet Bekiroglu, James Brown, Simon Rogers, Richard Shaw
- Aneurin Bevan University Health Board: Richard Parkin
- Barking, Havering and Redbridge University Hospitals NHS Trust: Neil Shah
- Barts Health NHS Trust (this now includes Whipps Cross University Hospital NHS Trust): Rishi Bhandari, Leo Cheng, Peter Hardee, Iain Hutchison, Simon Whitley
- Brighton and Sussex University Hospitals NHS Trust: James Herold, Michael Monteiro, Christian Surwald, John Weighill
- City Hospitals Sunderland NHS Foundation Trust: Andrew Burns, Simon Endersby, Ian Martin
- Derby Teaching Hospitals NHS Foundation Trust: Keith Jones, David Laugharne, Simon Lou
- East and North Hertfordshire NHS Trust: Andrew Camilleri
- Luton and Dunstable Hospital NHS Foundation Trust: Chi-Hwa Chan
- NHS Ayrshire & Arran: Stuart Hislop
- NHS Forth Valley: Joseph McManners
- North Cumbria University Hospitals NHS Trust: Graham Putnam
- Northampton General Hospital NHS Trust: Bill Smith
- Nottingham University Hospitals NHS Trust: Iain McVicar
- Portsmouth Hospitals NHS Trust: Peyman Alam, Rajiv Anand, John Blythe, Peter Brennan, Tim Mellor, Basavaiah Natesh, Clive Pratt
- Royal Free London NHS Foundation Trust: Jonathan Collier, Wayne Halfpenny, Janavikulam Thiruchelvam, Zaid Sadiq, Kaveh Shakib
- Royal Surrey County Hospital NHS Foundation Trust: Malcolm Bailey, Mike Bater, Martin Danford, Jacob D’Souza, Paul Johnson, Cyrus Kerawala
- South Tees Hospitals NHS Foundation Trust: Douglas Bryant, Colin Edge, Clarence Pace
- St George's University Hospitals NHS Foundation Trust: Kavin Andi, Nicholas Hyde, Graham Smith
- The Pennine Acute Hospitals NHS Trust: Wesam Aleid, Andrew Baldwin, Ewen Thomson, Bob Woodwards
- The Royal Wolverhampton NHS Trust: Nick Grew
- Torbay and South Devon NHS Foundation Trust: David Cunliffe, Lynne Fryer
- University College London Hospitals NHS Foundation Trust: Nicholas Kalavrezos, Colin Liew
- University Hospital of South Manchester NHS Foundation Trust: Mazhar Iqbal, Manu Patel
- University Hospitals Birmingham NHS Foundation Trust: Andrew Brown, Tim Martin, Hisham Mehanna, Satyesh Parmar, Paul Pracy, Prav Praveen, Keith Webster
- University Hospitals of Leicester NHS Trust: Chris Avery, Jonathan Hayter

**List of Tables and Figures**

| **Tables** |  |
| --- | --- |
| Supplementary Table 1 | Pathology assessment of the primary mouth tumour at baseline, and also of the neck nodes at baseline among those who had an elective neck dissection (randomised and observational cohort patients) |
| Supplementary Table 2 | Types of first events (for disease-free survival); randomised and observational cohort patients |
| Supplementary Table 3 | Maximum grade of adverse events for the randomised patients |
| Supplementary Table 4 | Details of adverse events for randomised patients |
| Supplementary Table 5 | Summary of adverse events for randomised patients with T1 tumours only |
| Supplementary Table 6 | Further treatments in randomised patients, after the initial surgical procedure (at baseline) |
| Supplementary Table 7 | Summary of adverse events for the observational cohort patients |
| Supplementary Table 8 | Trial designs of all 6 randomised studies of elective neck dissection for early stage OSCC |
| Supplementary Table 9 | Descriptive analyses comparing pathological features of the mouth tumour at baseline, or subsequent neck disease, between patients who had a recurrence/occurrence in the neck, and those who had no disease recurrence or other cancer nor died (randomised patients) |
|  |  |
| **Figures** |  |
| Supplementary Figure 1 | CONSORT diagram |
| Supplementary Figure 2 | Forest plot for subgroup analyses: overall survival, per-protocol analyses |
| Supplementary Figure 3 | Forest plot for subgroup analyses: disease-free survival, per-protocol analyses |
| Supplementary Figure 4 | Adjusted Kaplan-Meier curves among the observational cohort after adjustment for patient and tumour pathology factors |
| Supplementary Figure 5 | EORTC-QLQC30 health-related quality of life at 6 months post-surgery (compared to baseline) |
| Supplementary Figure 6 | EORTC-QLQ head & neck cancer specific health-related quality of life at 6 months post-surgery (compared to baseline) |

**Supplementary Table 1. Pathology assessment of the primary mouth tumour and tissue from the neck in those who had END, obtained from the initial surgery (assessment done by the local pathologist, as per routine practice)**

|  | SEND (randomised patients) | | | |  | | SEND (not randomised)^2^ | | | | |
| --- | --- | --- | --- | --- | --- | --- | --- | --- | --- | --- | --- |
|  | Resection only  N=124 | | Neck dissection^1^  N=126 | |  | | Resection only  N=234 | | | Neck dissection^1^  N=112 | |
|  | |  | |  | |  | |  |  | |  |
| pT-stage | |  | |  | |  | |  |  | |  |
| T0^6^ | | 1 (0.8) | | 1 (0.8) | |  | | 4 (1.7) | 1 (0.9) | |  |
| T1 | | 85 (68.5) | | 85 (67.5) | |  | | 177 (75.6) | 59 (52.7) | |  |
| T2 | | 27 (21.8) | | 26 (20.6) | |  | | 35 (15.0) | 40 (35.7) | |  |
| T3 | | - | | 2 (1.6) | |  | | 2 (0.8) | 2 (1.8) | |  |
| T4 | | 1 (0.8) | | 1 (0.8) | |  | | 3 (1.3) | 2 (1.8) | |  |
| TX | | 2 (1.6) | | 2 (1.6) | |  | | 13 (5.6) | 8 (7.1) | |  |
| Microinvasive | | 1 (0.8) | | 1 (0.8) | |  | | - | - | |  |
| Residual | | 1 (0.8) | | - | |  | | - | - | |  |
| Not stated | | 6 (4.8) | | 8 (6.4) | |  | | - | - | |  |
|  | |  | |  | |  | |  |  | |  |
| Invasive front | |  | |  | |  | |  |  | |  |
| Cohesive | | 29 (23.4) | | 29 (23.0) | |  | | 52 (22.2) | 17 (15.2) | |  |
| Non-cohesive | | 40 (32.3) | | 52 (41.3) | |  | | 60 (25.6) | 46 (41.1) | |  |
| Unknown | | 55 (44.4) | | 45 (35.7) | |  | | 122 (52.1) | 49 (43.8) | |  |
|  | |  | |  | |  | |  |  | |  |
| Completeness of resection | |  | |  | |  | |  |  | |  |
| Involved margins | | 22 (17.7) | | 13 (10.3) | |  | | 29 (12.4) | 9 (8.0) | |  |
| Margins <5mm | | 44 (35.5) | | 45 (35.7) | |  | | 87 (37.2) | 60 (53.6) | |  |
| Margins ≥5mm | | 46 (37.1) | | 61 (48.4) | |  | | 97 (41.5) | 38 (33.9) | |  |
| Unknown | | 12 (9.7) | | 7 (5.6) | |  | | 21 (9.0) | 5 (4.5) | |  |
|  | |  | |  | |  | |  |  | |  |
| Necrosis | | 40 (32.3) | | 30 (23.8) | |  | | 62 (26.5) | 38 (29.7) | |  |
| Perineural invasion | | 15 (12.1) | | 24 (19.0) | |  | | 31 (13.2) | 41 (36.6) | |  |
| Vascular/lymphatic invasion | | 11 (8.9) | | 6 (4.8) | |  | | 12 (5.1) | 16 (14.3) | |  |
| Bone invasion | | 5 (4.0) | |  | |  | | 2 (0.8) | 1 (0.9) | |  |
| Severe dysplasia | | 55 (44.4) | | 64 (50.8) | |  | | 106 (45.3) | 45 (40.2) | |  |
| Mild dysplasia at margin | | 9 (7.3) | | 7 (5.6) | |  | | 15 (6.4) | 6 (5.4) | |  |
| Moderate dysplasia at margin | | 12 (9.7) | | 10 (7.9) | |  | | 19 (8.1) | 3 (2.7) | |  |
| Severe dysplasia at margin | | 11 (8.9) | | 13 (10.3) | |  | | 25 (10.7) | 5 (4.5) | |  |
| Extra capsular spread (neck dissection only) | | - | | 10 (7.9) | |  | | - | 15 (13.4) | |  |
|  | |  | |  | |  | |  |  | |  |

1. Plus resection of the primary mouth tumour

2. Eligible patients approached for the trial but declined to participate, or were not approached for the trial (e.g. unavailability of surgeon, surgeon’s preference for one surgical procedure).

**Supplementary Table 2. Type of first events (for disease-free survival) among the SEND randomised patients (and what they were allocated to), and the prospective cohort (and what they received)**

|  | Resection only | Resection and neck dissection |
| --- | --- | --- |
| *Randomised patients* |  |  |
| New primary in mouth^1^ | 6 | 8 |
| Recurrence in mouth^2^ | 4 | 5 |
| **Occurrence/recurrence in neck** | **31^3,4^** | **14^5^** |
| **Recurrence in mouth & in neck** | **1^4^** | **5^5^** |
| New primary in mouth & in pharynx | 0 | 1 |
| New primary in throat/pharynx | 1 | 0 |
| Distant metastases | 0 | 1 |
| New primary not in the mouth, neck or pharynx | 4 | 3 |
| Death from oral cancer | 4 | 4 |
| Death from other causes | 13^6^ | 4^7^ |
| **Total** | **64** | **45** |
|  |  |  |
| *Observational cohort* |  |  |
| New primary in mouth^1^ | 4 | 0 |
| Recurrence in mouth^2^ | 10 | 2 |
| **Occurrence/recurrence in neck** | **43** | **10** |
| **Recurrence in mouth & in neck** | **6** | **4** |
| Recurrence in mouth & distant metastases | 0 | 1 |
| New primary in throat/pharynx | 1 | 0 |
| Distant metastases | 2 | 0 |
| New primary not in the mouth, neck or pharynx | 10 | 5 |
| Death from oral cancer | 13 | 11 |
| Death from other causes | 24 | 8 |
| **Total** | **113** | **41** |

1. A new malignancy not arising from the original tumour bed.

2. The same malignancy arising from the original tumour bed

3. For patients who developed neck nodes when they did not have neck dissection we refer to this as occurrence

4. All 31+1 patients had resection only

5. 4 of the 14 and 1 of the 5 patients actually had resection only and not with a neck dissection

6. Bacterial peritonitis/alcoholic-related liver disease; colon cancer; lung cancer (2); bronchopneumonia (n=2); bleeding due to oesophageal varices; diffuse large B cell lymphoma; suicide; coronary heart disease (n=2); COPD; unknown (possibly COPD)

7. Coronary heart disease (n=2); fire-related accident, renal disease

**Supplementary Table 3. Maximum grade of any adverse event in the randomised patients (number of patients, %)**

|  |  |  |
| --- | --- | --- |
| Maximum grade | Resection only  N=124 (%) | Neck dissection & resection  N=126 (%) |
| 0 | 49 (39.5) | 28 (22.2) |
| 1 | 29 (23.4) | 29 (23.0) |
| 2 | 24 (19.4) | 40 (31.7) |
| 3 | 14 (11.3) | 18 (14.3) |
| 4 | 4 (3.2) | 7 (5.6) |
| Not known | 4 (3.2) | 4 (3.2) |
| **Any grade 1-4*** | **75 (60.5)** | **98 (77.8)** |
|  |  |  |

*including not known (where there was a specific reported event, but the grade was missing). P=0.003 for the comparison

**Supplementary Table 4. Details of reported adverse events among randomised patients (based on the maximum grade for each patient and each event type), occurring up to 6 months of the initial surgery**

|  |  |  |  |  |  |  |  |
| --- | --- | --- | --- | --- | --- | --- | --- |
|  | Resection only  N=124 (%) | | |  | Neck dissection & resection  N=126 (%) | | |
|  | Grade unknown | Grade 1-2 | Grade 3-4^3^ |  | Grade unknown | Grade 1-2 | Grade 3-4^3^ |
|  |  |  |  |  |  |  |  |
| Mental nerve damage | 1 (0.8) |  |  |  | 1 (0.8) | 1 (0.8) |  |
| Lingual nerve damage |  | 11 (8.9) |  |  |  | 16 (12.7) |  |
| Hypoglossal nerve damage | 1 (0.8) | 1 (0.8) |  |  |  |  |  |
| Facial nerve damage |  | 1 (0.8) |  |  |  | 8 (6.4) |  |
| Cervical nerve damage |  | 2 (1.6) |  |  | 2 (1.6) | 7 (5.6) |  |
| Accessory nerve damage |  | 3 (2.4)^7^ | 1 (0.8)* ^7^ |  | 3 (2.4) | 23 (18.2) | 6 (4.8) |
| **Any of the above** |  | **17 (13.7)** | **1 (0.8)** |  |  | **43 (34.9)** | **6 (4.8)** |
|  |  |  |  |  |  |  |  |
| Difficulty chewing |  | 3 (2.4) |  |  |  | 2 (1.6) | 1 (0.8) |
| Poor tongue movement |  | 1 (0.8) |  |  |  |  | 1 (0.8) |
| Tethered tongue/periodontal disease |  | 1 (0.8) |  |  | 1 (0.8) | 4 (3.2) |  |
| Other eating problems |  | 1 (0.8) |  |  |  | 1 (0.8) |  |
| Mouth or tooth pain | 5 (4.0) | 3 (2.4) |  |  | 4 (3.2) | 8 (6.4) | 2 (1.6) |
| Saliva control problems |  | 1 (0.8)^14^ |  |  |  |  |  |
| Oral mucosal lesions or ulcers |  | 13 (10.5) | 1 (0.8) |  | 2 (1.6) | 8 (6.4) |  |
| Problem wearing denture |  | 1 (0.8) |  |  |  |  |  |
| Various other mouth issues |  | 2 (1.6) | 1 (0.8)^2^ |  | 2 (1.6) | 1 (0.8) |  |
| **Any of the above** |  | **26 (20.9)** | **2 (1.6)** |  |  | **28 (22.2)** | **3 (2.4)** |
|  |  |  |  |  |  |  |  |
| Dysphagia |  | 7 (5.5) | 1 (0.8) |  | 4 (3.2) | 9 (7.1) | 2 (1.6)* |
| Problem with naso-gastric tube |  | 1 (0.8) | 1 (0.8)* |  | 1 (0.8) | 9 (7.0) | 3 (2.3) |
| Tracheostomy |  |  |  |  |  |  | 2 (1.6)#^4^ |
| **Any of the above** |  | **8 (6.4)** | **2 (1.6)** |  |  | **19 (15.1)** | **6 (4.8)** |
|  |  |  |  |  |  |  |  |
| Speech problems |  | 7 (5.6) | 2 (1.6) |  | 1 (0.8) | 6 (4.8) |  |
| Vocal cord damage |  |  |  |  |  | 2 (1.6) |  |
| **Any of the above** |  | **7 (5.6)** | **2 (1.6)** |  |  | **9 (7.1)** |  |
|  |  |  |  |  |  |  |  |
| Swollen submandibular glands | 1 (0.8) | 8 (6.4) | 1 (0.8)* |  |  | 2 (1.6) | 1 (0.8)* |
| Swollen lymph glands |  | 2 (1.6) |  |  | 1 (0.8) |  |  |
| Swollen parotid gland |  | 1 (0.8) |  |  |  |  |  |
| Inflammation of neck or face |  | 5 (4.0) | 2 (1.6) |  | 1 (0.8) | 8 (6.3) | 3 (2.4) |
| Sore throat/tonsillitis | 1 (0.8) | 1 (0.8) |  |  |  | 3 (2.4) |  |
| **Any of the above** |  | **18 (14.5)** | **2 (1.6)** |  |  | **15 (11.9)** | **4 (3.2)** |
|  |  |  |  |  |  |  |  |
| Scarring (local resection site) | 3 (2.4) |  | 1 (0.8) |  |  | 2 (1.6) |  |
| Wound healing problems^1^ | 1 (0.8) | 6 (4.8) |  |  |  | 7 (5.6) | 1 (0.8)* |
| Wound problems (neck dissection) |  | 1 (0.8)^6^ | 1 (0.8)^6^ |  |  | 3 (2.4) | 2 (1.6) |
| Free flap reconstruction issues |  |  |  |  |  | 3 (2.4) |  |
| **Any of the above** |  | **10 (8.1)** | **2 (1.6)** |  |  | **14 (11.1)** | **3 (2.4)** |
|  |  |  |  |  |  |  |  |
| Other events likely associated with surgery: | |  |  |  |  |  |  |
| Haemorrhage or haematoma |  | 9 (7.3)^5^ | 6 (4.8)#^5^ |  | 2 (1.6)^5^ | 10 (7.9)^5^ | 5 (4.0)$^5^ |
| Pain (unspecified, or not known to be in mouth, neck, arm or shoulder) | 1 (0.8) | 11 (8.9) | 4 (3.2) |  | 3 (2.4) | 28 (22.2) | 4 (3.2)* |
| Problems with hearing or taste |  | 1 (0.8) |  |  |  | 3 (2.4) | 1 (0.8) |
| Mandible fracture |  |  |  |  |  |  | 1 (0.8) |
| Urinary problems |  | 1 (0.8)^8^ |  |  | 3 (2.3)^8^ | 1 (0.8)^8^ |  |
|  |  |  |  |  |  |  |  |
| Possibly chemo/radiotherapy related: |  |  |  |  |  |  |  |
| Anorexia or weight loss | 1 (0.8) | 1 (0.8) | 1 (0.8) |  |  | 3 (2.3)^12^ | 1 (0.8) |
| Diarrhoea |  | 1 (0.8) |  |  |  | 1 (0.8) |  |
| Nausea or vomiting^13^ | 1 (0.8) | 3 (2.4) | 1 (0.8) |  | 1 (0.8) | 7 (5.6)^13^ | 1 (0.8) |
| Skin problems |  | 4 (3.2)^11^ |  |  | 2 (1.6)^11^ | 5 (4.0)^11^ |  |
| Blood levels (abnormal) | 1 (0.8) |  |  |  |  | 2 (1.6) |  |
| Dry mouth |  | 3 (2.4)^9^ |  |  | 1 (0.8) | 3 (2.4)^9^ |  |
| Limited mouth opening | 1 (0.8)^10^ | 8 (6.4) | 2 (1.6) |  | 2 (1.6)^10^ | 4 (3.2)^10^ | 1 (0.8) |
| **Any of the above** |  | **19 (15.3)** | **3 (2.4)** |  |  | **22 (17.5)** | **2 (1.6)** |
|  |  |  |  |  |  |  |  |
| Chest problems |  | 1 (0.8) | 1 (0.8)* |  | 1 (0.8) | 4 (3.2) | 3 (2.4)# |
| Chest infection/septicaemia | 1 (0.8) | 1 (0.8) |  |  |  | 1 (0.8) | 2 (1.6)* |
| Fatigue or depression |  | 1 (0.8) | 3 (2.4) |  | 3 (2.4) | 4 (3.2) |  |
| Heart problems | 1 (0.8) |  |  |  |  | 1 (0.8) | 1 (0.8) |
| Other gastrointestinal (not above) |  | 2 (1.6) |  |  | 3 (2.4) |  |  |
| Probably venous thrombosis |  |  |  |  | 1 (0.8) | 1 (0.8) | 1 (0.8) |
| Stomach ulcers/vomiting blood |  | 1 (0.8) | 2 (1.6)* |  |  | 1 (0.8) |  |
| Other | 2 (1.6) | 2 (1.6) | 3 (2.4)* |  | 1 (0.8) | 10 (7.9) | 2 (1.6) |
|  |  |  |  |  |  |  |  |
| Any adverse event (any grade) | 4 (3.2) | 53 (42.7) | 18 (14.5) |  | 4 (3.1) | 69 (54.8) | 25 (19.8) |
|  |  |  |  |  |  |  |  |

In each row above, a patient is only counted once.

For the ‘Any of the above’ rows a patient can move between columns in the table, hence the subtotal is not necessarily the sum of the individual components. Also, patients with unknown grade are included with grades 1-2.

Further treatments indicated below, were those reported to the trials office.

1. Slow healing, infection, or rupture (dehiscence)

2. Grade 3 lichen planus

3. All grade 3-4 adverse events were grade 3, except where indicated by * (n=1 had grade 4), # (n=2 had grade 4) or $ (n=3 had grade 4)

4. Not reported whether the tracheostomy was ongoing or not

5. Resection only: 5 of 6 patients with grade 3-4 occurred within 30 days post-operatively (n=4 within 3 days), 7 of 9 patients with grade 1-2 occurred within 11 days.

Neck dissection: 4 of 5 patients with grade 3-4 occurred within 27 days post-operatively (n=3 within 3 days), 8 of 10 patients with grade 1-2 occurred within 13 days, and 2 of 2 patients with unknown grade occurred within 15 days.

6. The patient with grade 3 had a neck dissection+resection instead of the assigned resection. The other event also appeared to be due to subsequent neck dissection (though later surgery was not reported to the trials office)

7. Of these 4 patients, 2 had a neck dissection+resection instead of the assigned resection only (including the grade 4 event). There is no record of neck dissection for the two others

8. Resection only: the event occurred post-operatively but there is no record of having had a neck dissection

Neck dissection: all 4 cases were post-operative.

9. Resection only: n=2 (grade 2) had RT alone up to 3.2 months before the adverse event date.

Neck dissection: n=2 (grade 1 & 2) had RT alone up to 4.7 months before the event

10. Resection only: n=1 with unknown grade had ChemoRT 3.4 months before the event date.

Neck dissection: n=2 with unknown grade had RT alone 3.2 months before the event; n=1 with grade 2 had ChemoRT 4.1 months before

11. Resection only: all 4 had RT alone (n=3 up to 3.1 months before, the other unknown).

Neck dissection: of the 2 with unknown grade n=1 had RT alone and n=1 had ChemoRT (both up to 0.8 months before); and of those with grade 2, n=2 had RT alone up to 1.1 months before

12. Neck dissection: n=1 with grade 1 had ChemoRT 1.4 months before. None of the resection group were recorded as having chemotherapy

13. Neck dissection: n=1 with grade 1 had ChemoRT just before the event. No other patient in either trial group were recorded as having chemotherapy

14. Occurred post-operatively

RT=radiotherapy

ChemoRT=chemo-radiotherapy

**Supplementary Table 5. Adverse events among randomised patients occurring within 6 months of the baseline surgery (based on the maximum grade for each patient and each event type) – only for patients with clinical stage T1 tumours.**

|  |  |  |  |  |  |  |
| --- | --- | --- | --- | --- | --- | --- |
|  | Resection only  N=80 (%) | |  | Neck dissection & resection  N=79 (%) | | P-value* |
|  | Grade 1-2/ unknown | Grade 3-4 |  | Grade 1-2/ unknown | Grade 3-4 |  |
| Nerve damage (face or neck) | 9 (11.2) | 1 (1.2) |  | 32 (40.5) | 3 (3.8) | P<0.001 |
| Problems in mouth | 13 (16.2) | 2 (2.5) |  | 18 (22.8) | 0 | P=0.24 |
| Problems swallowing | 2 (2.5) | 1 (1.2) |  | 13 (16.5) | 0 | P=0.004 |
| Speech/vocal cord problems | 0 | 0 |  | 7 (8.9) | 0 | P=0.006 |
| Swollen glands/swelling in mouth/neck | 12 (15.0) | 0 |  | 11 (13.9) | 3 (3.8) | P=0.33 |
| Problems taste/hearing |  |  |  | 2 (2.5) | 1 (1.3) | P=0.12 |
| Wound healing problems | 9 (11.2) | 1 (1.2) |  | 8 (10.3) | 3 (3.8) | P=0.68 |
| Possibly related to chemo/RT^1^ | 13 (16.2) | 2 (2.5) |  | 11 (13.9) | 1 (1.3) | P=0.81 |
|  |  |  |  |  |  |  |
| Any event# | 40 (50.0) | 10 (12.5) |  | 47 (59.5) | 14 (17.7) | P=0.12 |
|  |  |  |  |  |  |  |

*Chi-squared or Fisher’s exact test for comparing the two treatment groups

# Any adverse event recorded, and each patient only counted once

1. Weight loss, diarrhoea, nausea/vomiting, skin rash, abnormal biochemistry, dry mouth, and limited mouth opening after radiotherapy

**Supplementary Table 6. Further treatments given, according to the trial arm to which patients were randomly allocated (RCT), or the surgical procedure received (observational cohort)**

|  | Randomised patients | |  | Observational cohort | |
| --- | --- | --- | --- | --- | --- |
|  | Resection only  N=124 | Neck dissection & resection  N=126 |  | Resection only  N=234 | Neck dissection & resection  N=112 |
|  |  |  |  |  |  |
|  | Number of patients (%) | |  | Number of patients (%) | |
| Surgery for complications: | 10 (8.1) | 8 (6.3) |  | 7 (3.0) | 14 (12.5) |
|  |  |  |  |  |  |
| Neck dissection (with or without resection)* | 24 (19.3)  (23 post-recurrence; 15 died)^3^ | 16 (12.7)  (16 post-recurrence; 12 died)^3^ |  | 60 (25.6)  (33 post-recurrence)^3^ | 9 (8.0)  (9 post-recurrence)^3^ |
| Median time from initial surgery (range), months | 9.1 (1.1-19.4) | 13.2 (3.3-35.7) |  |  |  |
|  |  |  |  |  |  |
| Local resection only (patients had no neck dissection at any time)* | 14 (11.3)  (9 post-recurrence; 3 died)^3^ | 10 (7.9)  (5 post-recurrence; 2 died)^3^ |  | 33 (14.1)  (4 post-recurrence)^3^ | 4 (3.6) |
| Median time from initial surgery (range), months | 18.3 (0.5-43.0) | 3.0 (0.5-43.2) |  |  |  |
|  |  |  |  |  |  |
| Excision biopsy* | 13 (10.5) | 9 (7.1) |  | 10 (4.3) | 6 (5.4) |
|  |  |  |  |  |  |
| Radiotherapy alone^1^ | 22 (17.7) | 19 (15.1) |  | 33 (14.1) | 36 (32.1) |
| Chemotherapy alone^1^ | 4 (3.2) | 6 (4.8) |  | 0 | 1 (0.9) |
| Chemo-radiotherapy^1^ | 11 (8.9) | 10 (7.9) |  | 13 (5.5) | 13 (11.6) |
| *Any chemotherapy or radiotherapy^1^* | *32 (25.8)* | *30 (23.8)* |  | *44 (18.8)* | *47 (42.0)* |
| *Any chemotherapy or radiotherapy^2^* | *24 (19.3); 17 died* | *13 (10.3); 11 died* |  | *31 (13.2)* | *11 (9.8)* |
|  |  |  |  |  |  |
| Palliative care | 2 | 3 |  | 4 | 3 |

*not part of the initial planned procedure at baseline (i.e. these were genuine further interventions, for example, when involved margins were found, which could occur after the pathology examination following the initial neck dissection).

All chemotherapy was platinum-based.

1. At any time

2. Post-recurrence

3. Known to have been post-recurrence (uncertainty over the others)

In Table 1 there are 29 patients who had a neck dissection, and N1 or N2 disease (randomised trial); 13 of these 29 were known to have received adjuvant radiotherapy or platinum chemo-radiotherapy. Of the 10 patients who had extra capsular spread (Supplementary Table 1), 7 were known to have received radiotherapy or chemo-radiotherapy 1.5-4 months after the initial surgery. Among the 38 patients who had a neck dissection, and N1 or N2 disease (observational cohort); 20 were known to have received adjuvant radiotherapy or platinum chemo-radiotherapy. Of the 15 patients who had extra capsular spread (Supplementary Table 1), 13 were known to have received radiotherapy or chemo-radiotherapy.

**Supplementary Table 7. Adverse events among the observational (real world) cohort occurring up to 6 months from the baseline surgery (based on the maximum grade for each patient and each event type)**

|  |  |  |  |  |  |  |
| --- | --- | --- | --- | --- | --- | --- |
|  | Resection only  N=234 (%) | |  | Neck dissection & resection  N=112 (%) | | P-value (Fisher’s exact test) |
|  | Grade 1-2/ unknown | Grade 3 |  | Grade 1-2/ unknown | Grade 3 |  |
| Nerve damage (face or neck) | 34 (14.5) |  |  | 24 (21.4) |  | P=0.12 |
| Problems in mouth | 16 (6.8) | 3 (1.3) |  | 16 (14.3) | 3 (2.7) | P=0.04 |
| Problems swallowing | 3 (1.3) | 1 (0.4) |  | 9 (8.0) | 3 (2.7) | P<0.001 |
| Speech/vocal cord problems | 11 (4.7) |  |  | 8 (7.1) |  | p=0.45 |
| Swollen glands/swelling in mouth or neck | 13 (5.6) |  |  | 7 (6.2) |  | P=0.81 |
| Problems taste/hearing | 1 (0.4) |  |  |  | 1 (0.9) | P=0.54 |
| Wound healing problems | 6 (2.6) | 1 (0.4) |  | 10 (8.9) | 3 (2.7) | P=0.006 |
| Possibly related to chemo/RT^1^ | 12 (5.1) | 1 (0.4) |  | 10 (8.9) | 2 (1.8) | P=0.13 |
|  |  |  |  |  |  |  |
| Any event recorded (each patient counted once) | 85 (36.3) | 8 (3.4) |  | 54 (48.2) | 14 (12.5) | P<0.001 |
|  |  |  |  |  |  |  |

There were no grade 4 events

1. Weight loss, diarrhoea, nausea/vomiting, skin rash, abnormal biochemistry, dry mouth, and limited mouth opening after radiotherapy

**Supplementary Table 8. Characteristics of all randomised trials that have evaluated elective neck dissection for early stage OSCC.**

**Patients in all trials except one had surgical resection of the primary mouth tumour, and in the other trial they had interstitial radiotherapy only for the mouth tumour.^10^**

| First author (reference) | Location | Recruitment dates | Cancer site | No. patients | Average age (years) | Sex* | Stage | Differentiation |
| --- | --- | --- | --- | --- | --- | --- | --- | --- |
|  |  |  |  |  |  |  |  |  |
| Vandenbrouck 1980^10^ | Paris, France  (1 hospital) | 1966-1973 | Oral cavity  Tongue 55%  Floor mouth 45% | 75 | 57 | M 89%  F 11% | T1 20%  T2 64%  T3 13%  Unknown 3% | Well 87%  Poorly 9%  Undifferentiated 4% |
| Fakih 1989^11^ | Mumbai, India  (1 hospital) | 1985-1988 | Tongue | 70 | ~45 | M 64%  F 36% | T1 34%  T2 66% | Not reported |
| Kligerman 1994^12^ | Rio de Janeiro, Brazil  (1 hospital) | 1987-1992 | Oral cavity  Tongue 61%  Floor mouth 39% | 67 | 57 | M 78%  F 22% | T1 46%  T2 54% | Well 30%  Moderate 70% |
| Yuen 2009^13^ | Hong Kong  (3 hospitals) | 1996-2004 | Tongue | 71 | 57 | M 61%  F 39% | T1 61%  T2 39% | Well 47%  Moderate 42%  Poorly 11% |
| D’Cruz 2015^14^ | Mumbai, India  (1 hospital) | 2002-2014 | Oral cavity  Tongue 85%  Floor mouth 1%  Buccal mucosa 14% | 596  (results for 496) | 48 | M 75%  F 25% | T1 44%  T2 56% | Well/moderate 78%  Poorly 5%  Unknown 5% |
| SEND (current) | UK  (25 hospitals) | 2007-2015 | Oral cavity  Tongue 61%  Floor mouth 19%  Buccal mucosa 10%  Other 10% | 250 | 63 | M 64%  F 36% | T1 64%  T2 36% | Well 13%  Moderate 57%  Poorly 21%  Unknown 9% |
|  |  |  |  |  |  |  |  |  |

*M:male, F:female

**Supplementary Table 9. Randomised patients: comparison of pathological features of the mouth tumour or neck disease at baseline, or neck disease at recurrence, between patients who had a recurrence/occurrence in the neck, and those who had no disease recurrence or other cancer nor died, according to the surgery they actually received at baseline. The table shows the percentages of the number of patients in each column (they do not always sum to 100 because the denominators include patients with unknown results, which are not shown in the table)**

|  | Resection only | |  | Neck dissection & resection | |
| --- | --- | --- | --- | --- | --- |
|  | Alive & disease-free  N=58 | Neck  occurrence  N=37* |  | Alive &  disease-free  N=82 | Neck  recurrence  N=14* |
| Differentiation |  |  |  |  |  |
| Poorly | 13.8 | 32.4 |  | 19.5 | 50.0 |
| Moderately | 60.3 | 54.0 |  | 57.3 | 50.0 |
| Well | 15.5 | 5.4 |  | 13.4 | 0 |
|  |  |  |  |  |  |
| pTStage |  |  |  |  |  |
| Microinvasive | 1.7 |  |  | 1.2 |  |
| pT0 |  |  |  | 1.2 |  |
| pT1 | 79.3 | 54.0 |  | 75.6 | 28.6 |
| pT2 | 10.3 | 35.1 |  | 18.3 | 64.3 |
| pT3 or 4 |  |  |  | 1.2 | 7.1 |
|  |  |  |  |  |  |
| Invasive front |  |  |  |  |  |
| Cohesive | 19.0 | 24.3 |  | 18.8 | 21.4 |
| Not cohesive | 29.3 | 51.3 |  | 37.6 | 64.3 |
|  |  |  |  |  |  |
| Completeness of resection |  |  |  |  |  |
| Involved margins | 15.5 | 10.8 |  | 12.2 | 7.1 |
| Margins <5mm | 32.8 | 54.0 |  | 28.0 | 64.3 |
| Margins ≥5mm | 43.1 | 29.7 |  | 54.9 | 21.4 |
|  |  |  |  |  |  |
| Necrosis | 32.8 | 37.8 |  | 24.4 | 50.0 |
| Perineural invasion | 8.6 | 27.0 |  | 19.5 | 35.7 |
| Vascular/lymphatic invasion | 5.2 | 13.5 |  | 3.7 | 21.4 |
| Bone invasion | 0 |  |  | 3.7 |  |
| Severe dysplasia | 48.3 | 37.8 |  | 46.3 | 42.9 |
| Mild dysplasia at margin | 3.4 | 0 |  | 3.7 | 14.3 |
| Moderate dysplasia at margin | 5.2 | 8.1 |  | 6.1 | 7.1 |
| Severe dysplasia at margin | 13.8 | 5.4 |  | 7.3 | 0 |
|  |  |  |  |  |  |
|  | Median values shown for these 4 factors | | | | |
| Maximum tumour diameter (mm) by pathology | 9.5 | 17.5 |  | 13.2 | 24.5 |
| Depth of invasion (mm) | 3.5 | 7.0 |  | 4.5 | 8.5 |
| Mucosal margin (mm) | 5.0 | 4.2 |  | 5.0 | 5.0 |
| Deep margin (mm) | 5.0 | 5.0 |  | 5.0 | 3.0 |
|  |  |  |  |  |  |
| Pathological features of neck disease at baseline: | |  |  |  |  |
| pNstage |  |  |  |  |  |
| N0 (% of patients) |  |  |  | 82.9 | 42.9 |
| N1 |  |  |  | 14.6 | 21.4 |
| N2 |  |  |  | 2.4 | 35.7 |
| Extra capsular spread (% of patients) |  |  |  | 2.4% | 42.9% |
| % nodes removed that were positive, median (range) | |  |  | 0% (0-14.2) | 3.4% (0-13.6) |
| Largest positive node mm, median (range) |  |  |  | 5.6 (0.6-10) | 7.5 (4-12) |
|  |  |  |  |  |  |
| Pathological features of neck disease at recurrence/occurrence**: | |  |  |  |  |
| Extra capsular spread (% of patients) |  | 76% |  |  | 75% |
| % nodes removed that were positive, median (range) | | 9.0% (1.7-42) |  |  | 4.4,6.7,21.0,29.4% |
| Largest positive node mm, median (range) |  | 25 (6.6-60) |  |  | 9.5,17,30,53 |
|  |  |  |  |  |  |

*the 37+14 cases come from Supplementary Table B (first events that were disease in the neck, or mouth & neck: 31+1+14+5). Of these 51, 37 actually had resection only and the other 14 had a neck dissection at baseline. In Supplementary Table B, the 5 patients allocated to END who actually had resection only are added to the 32 in the resection only group, to give total of 37 above.

** based on 25 patients (resection only) and 4 patients (neck dissection plus resection) who had pathology data reported for the occurrence/recurrence disease (the individual data values are shown for % positive nodes and largest positive node for the latter surgical group).

**Supplementary Figure 1. CONSORT diagram (OS overall survival, DFS disease-free survival)**


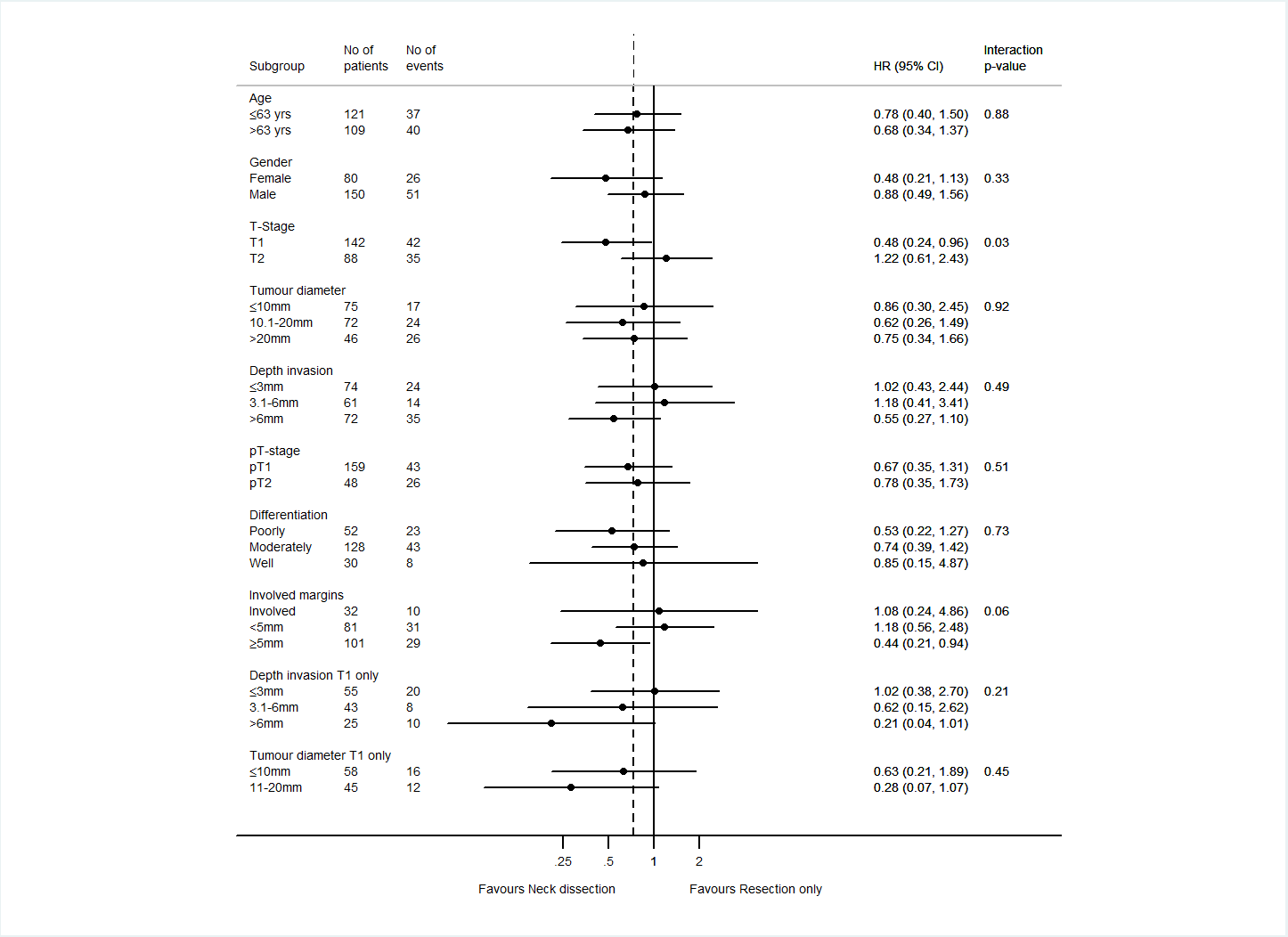


**Supplementary Figure 2. Subgroup analysis for overall survival (per-protocol). The vertical dashed line is the overall treatment effect for elective neck dissection (hazard ratio 0.65) [Tumour diameter by pathology].**

There was only a suggestion that the benefit for END appeared greater for clinical stage T1 than T2 tumours, but the interaction p-value was only marginally statistically significant (p=0.03). Importantly, the 95% CI for both T1 and T2 groups include the overall HR of 0.73.

The interaction p-value tests whether the HR differs between each subgroup. The dashed vertical line tests whether each subgroup HR differs from the overall treatment effect (ie from a HR of 0.80). They are two different ways of testing for subgroup effects (see Dehbi & Hackshaw JCO 2016; 35(2): 253-254. Investigating Subgroup Effects in Randomized Clinical Trials)


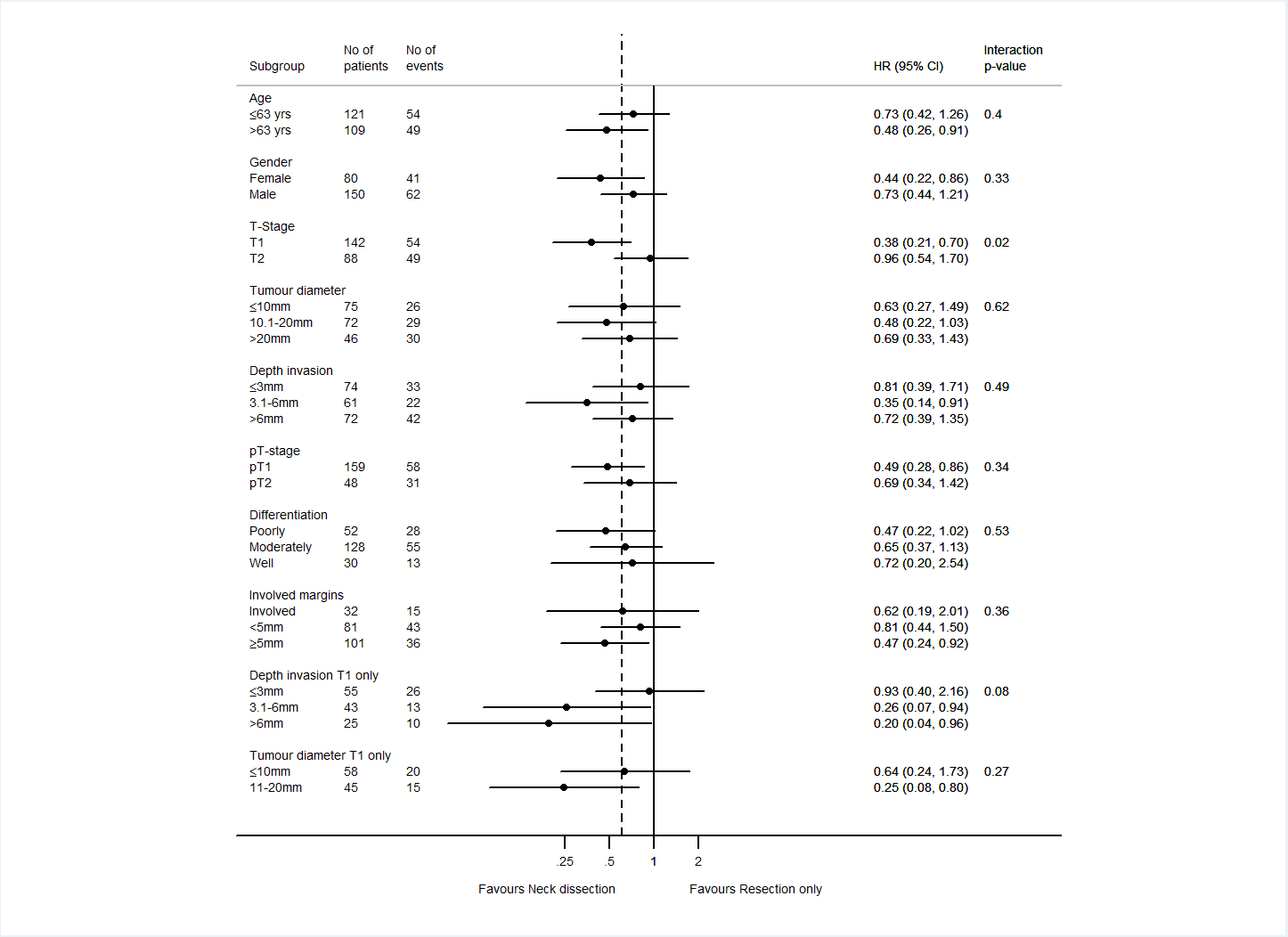


**Supplementary Figure 3. Subgroup analysis for disease-free survival (per-protocol). The vertical dashed line is the overall treatment effect for elective neck dissection (hazard ratio 0.61) [Tumour diameter by pathology].**

**Supplementary Figure 4. Adjusted Kaplan-Meier curves among the observational cohort. The left-hand side figures are adjusted for baseline patient factors (age, sex, smoking status, alcohol drinking status, geographical location, clinical T-stage, and site of tumour in the mouth). The right-hand side figures are adjusted for the same factors plus tumour pathology features (pathology assessed tumour diameter, depth of invasion, differentiation and completeness of resection)**

**Supplementary Figure 5. EORTC Quality of life score. The graph shows for each patient the 6 months score minus the baseline score. A positive difference indicates that QoL improved by 6 months; a negative difference that QoL worsened by 6 months. Median (25-75^th^ centile shown in red). P-values from a non-parametric Wilcoxon test.**

**Supplementary Figure 5 continued. EORTC Quality of life score (functional scales). The graph shows for each patient the 6 months score minus the baseline score. A positive difference indicates that QoL improved by 6 months; a negative difference that QoL worsened by 6 months. Median (25-75^th^ centile shown in red). P-values from a non-parametric Wilcoxon test.**

**Supplementary Figure 5 continued. EORTC Quality of life score (symptom scales). The graph shows for each patient the 6 months score minus the baseline score. A positive difference indicates that QoL worsened by 6 months; a negative difference that QoL improved by 6 months. Median (25-75^th^ centile shown in red). P-values from a non-parametric Wilcoxon test.**

**Supplementary Figure 5 continued. EORTC Quality of life score (symptom scales). The graph shows for each patient the 6 months score minus the baseline score. A positive difference indicates that QoL worsened by 6 months; a negative difference that QoL improved by 6 months. Median (25-75^th^ centile shown in red). P-values from a non-parametric Wilcoxon test.**

**Supplementary Figure 6. EORTC Quality of life score (symptom scales from the head&neck cancer module). The graph shows for each patient the 6 months score minus the baseline score. A positive difference indicates that QoL worsened by 6 months; a negative difference that QoL improved by 6 months. Median (25-75^th^ centile shown in red). P-values from a non-parametric Wilcoxon test.**

**Supplementary Figure 6 continued. EORTC Quality of life score (symptom scales from the head&neck cancer module). The graph shows for each patient the 6 months score minus the baseline score. A positive difference indicates that QoL worsened by 6 months; a negative difference that QoL improved by 6 months. Median (25-75^th^ centile shown in red). P-values from a non-parametric Wilcoxon test.**

**Supplementary Figure 6 continued. EORTC Quality of life score (symptom scales from the head&neck cancer module). The graph shows for each patient the 6 months score minus the baseline score. A positive difference indicates that QoL worsened by 6 months; a negative difference that QoL improved by 6 months. Median (25-75^th^ centile shown in red). P-values from a non-parametric Wilcoxon test.**
